# Endophytic *Fusarium commune* G3-29-Mediated dsRNA Delivery for Efficient Control of Western Flower Thrips

**DOI:** 10.3390/jof12040291

**Published:** 2026-04-18

**Authors:** Xueyuan Sheng, Yanfei Wang, Chang Chen, Chao Ma, Shuangchao Wang, Endong Wang, Yan Zhao, Lihua Guo

**Affiliations:** 1 Key Laboratory of National Forestry and Grassland Administration on Native Grass Breeding, College of Grassland Science, Inner Mongolia Agricultural University, Hohhot 010018, China; 2 State Key Laboratory for Biology of Plant Diseases and Insect Pests, Institute of Plant Protection, Chinese Academy of Agricultural Sciences, Beijing 100193, China

**Keywords:** western flower thrips, RNA interference, endophytic fungus, *Fusarium commune* G3-29, biological control

## Abstract

RNA interference (RNAi) provides a sequence-specific strategy for pest management, but efficient and stable double-stranded RNA (dsRNA) delivery remains a key challenge. Here, we established a plant-probiotic-based gene silencing system using the endophytic fungus *Fusarium commune* G3-29 as a dsRNA delivery vector against western flower thrips (WFTs, *Frankliniella occidentalis*). Recombinant G3-29 strains expressing dsRNA targeting the essential WFT genes *ACT* and *SNF* were constructed and confirmed to colonize kidney bean leaves without pathogenicity. Bioassays showed that feeding on leaves colonized by dsRNA-expressing G3-29 significantly decreased survival and downregulated target gene expression in both WFT larvae and adults. Within 4 days, survival of both larvae and adults fell below 10%. In larvae, target gene expression decreased by 63% (*ACT*) and 33% (*SNF*), while in adults, reductions of 74% (*ACT*) and 65% (*SNF*) were observed. In contrast, in vitro-synthesized dsRNA failed to induce significant gene silencing or mortality in larvae, and its control efficacy against adults was also inferior to that of endophytic fungus-mediated dsRNA delivery. Our findings establish endophytic fungus *F. commune* G3-29 as an effective and sustainable dsRNA delivery vehicle for RNAi-based pest control, offering distinct advantages over existing strategies such as HIGS and SIGS. This approach provides a promising new direction for managing WFTs and other insect pests.

## 1. Introduction

RNA interference (RNAi) is a highly conserved mechanism in most eukaryotes [1,2] and plays a critical role in normal development and physiological processes [3]. RNAi is triggered by the presence of double-stranded RNA (dsRNA), which leads to the sequence-specific degradation of complementary messenger RNA (mRNA) molecules [4]. The core process involves the introduction of exogenous dsRNA designed to target essential insect genes. This dsRNA is processed by the insect’s cellular machinery into small interfering RNAs (siRNAs), which then guide the RNA-induced silencing complex (RISC) to cleave corresponding target mRNAs, ultimately resulting in gene silencing and pest mortality [5,6].

At present, RNAi has emerged as a promising tool for pest control [5,7]. Unlike conventional agrochemicals, RNAi operates in a sequence-dependent manner via dsRNA, a naturally occurring molecule that offers distinct advantages in target specificity and efficacy [8]. The application of RNAi for pest management induces a spectrum of detrimental effects in target insects, including developmental retardation, diminished reproductive capacity, and increased mortality rates [9,10,11]. In laboratory settings, several methods, such as immersion, microinjection, and nanoparticle encapsulation, have been successfully used to deliver dsRNA and trigger RNAi responses in various pests [12,13,14,15]. However, the main obstacle to field application remains the efficient and stable delivery of dsRNA [16]. Although spray-induced gene silencing (SIGS) and host-induced gene silencing (HIGS) have shown promising application prospects in the field of pest control, their performance in terms of sustainability and the regulatory issues brought about by transgenic technology remain practical challenges for their application [17,18].

The western flower thrips (WFTs, *Frankliniella occidentalis*; Thysanoptera) is a major agricultural pest worldwide, renowned for its wide host range, strong reproductive capacity, and efficient virus transmission ability [19]. Native to North America, this insect has successfully invaded regions across the globe, posing a serious threat to diverse crops including vegetables, flowers, and cash crops [20,21]. The combination of their small size and the difficulties in detection and identification allows thrips invasions to often succeed undetected [22]. In addition, WFTs feed by injuring plant tissue, exposing the mesophyll, secreting enzymes to digest the ruptured tissue, and then sucking up the digested compounds. This feeding behavior causes silver leafing, significantly reduces photosynthesis, and creates conditions conducive to pathogen development on injured leaves [23]. Traditionally, the use of chemical pesticides to control WFTs has been the main strategy, such as the two major pesticides: broad-spectrum insecticides and narrow-spectrum insecticides [24]. However, long-term and excessive use of these chemicals has led to widespread resistance in thrips populations [25]. Therefore, RNAi technology, which achieves specific control by targeting essential pest genes, offers a promising new direction for addressing this challenge [16]. Recently, “Plant-Probiotic-Based Gene Silencing (PPGS)” has emerged as a novel RNAi-based control strategy, utilizing plant probiotics as the microbial chassis for dsRNA expression to achieve effective control of polyphagous pests [3].

Endophytes are the plant-associated microbes residing inside the plant tissue (inter- or intra-cellularly) without producing any negative impact or symptoms to the host plant [26]. In addition, endophytes also protect plants from abiotic and biotic stresses [27]. The fungal endophyte strain G3-29 is isolated from healthy wheat roots and identified as *Fusarium commune*. The fungal endophyte strain G3-29 is an important plant probiotic and exhibits strong antagonistic activity against fungal pathogens in vitro. Furthermore, it significantly protects wheat spikes from *Fusarium* head blight (FHB) in vivo and promotes plant growth [28]. Based on these characteristics, we hypothesize that leveraging the stable colonization ability of the endophytic fungus G3-29, it can be engineered to express pest-targeting dsRNA, forming the basis of a novel pest control strategy.

In this study, we explored the potential of the endogenous fungus *F. commune* G3-29 as a dsRNA delivery platform. The fungus was engineered to express dsRNA targeting the essential WFT genes, *Actin* (*ACT*) and transport III subunit *Snf7* (*SNF*) [15]. We confirmed that the recombinant G3-29 strain could successfully colonize host plant tissues and produce target dsRNAs. We then systematically evaluated this endophyte-mediated RNAi system for its ability to induce gene silencing in WFTs, providing a new approach for pest control.

## 2. Materials and Methods

### 2.1. Plant Material and WFT Rearing

WFT colonies were reared in tubular glass jars (2 L) containing surface-sterilized kidney beans and two-week-old kidney bean leaves. The jar openings were covered with fine mesh to provide adequate ventilation. All insects were maintained under controlled conditions at 25 ± 2 °C, 70 ± 10% relative humidity, and a 12 h light/12 h dark photoperiod (L12:D12). For synchronized collection of first-instar larvae or adults, female adults were allowed to oviposit on fresh kidney beans for 3 days. Beans containing eggs were then transferred to new glass jars and incubated until the desired developmental stage was reached.

### 2.2. Fungal Strains

We used the *F. commune* strain G3-29 as the original strain. In this study, for morphological observation, we cultured the fungus on potato dextrose agar (PDA) at 25 °C for 4–5 days. For RNA extraction, cultures were grown on cellophane-overlaid PDA plates. Conidia were obtained by shaking incubation in carboxymethyl cellulose (CMC) liquid medium at 25 °C for 4 days.

### 2.3. Plasmid Construction

To test the silencing effect of dsRNA on WFTs, the 321 bp sequence of the ACT gene and the 402 bp sequence of the SNF gene were cloned from the total RNA of WFTs by RT-PCR. Meanwhile, the GFP gene was cloned from the pGTN-GFP vector as a control and inserted into the pRP27-Eno1 vector digested with *BamH*I and *Spe*I. Finally, three dsRNA vectors pRP27-Eno1-GFP, pRP27-Eno1-ACT, and pRP27-Eno1-SNF were obtained.

### 2.4. Protoplast Preparation and Transfection Assays

We conducted protoplast preparation and plasmid transformation, as previously described [28]. Briefly, the G3-29 strain was cultured in the CMC medium to obtain conidia. Approximately 1 × 10^7^ conidia were cultured overnight in the Yeast Extract Peptone Dextrose liquid medium, and the mycelia were collected using three layers of sterile lens paper. To release protoplasts, fresh mycelia were treated with a digestive solution of 1.2 M KCl, 2.5% (*w*/*v*) driselase, 1% (*w*/*v*) lyticase, and 1% (*w*/*v*) snailase. Following digestion, the protoplasts were filtered through three layers of lens paper and subsequently washed with STC buffer (1 M sorbitol, 50 mM Tris-HCl, pH 8.0, 50 mM CaCl_2_∙2H_2_O).

During the transformation process, 5 µg (≈20 µL) of dsRNA plasmid was mixed with 400 µL of protoplasts and incubated on ice for 30 min. Then, 1.5 mL of PTC buffer (40% polyethylene glycol 4000 in STC buffer) was added dropwise to the mixture. The mixture was then incubated at room temperature for 30 min. Following this, 5 mL of liquid LB was added and shaken for overnight cultivation. Finally, the transformation mixture was spread onto TB3 plates containing the appropriate antibiotic for selection.

### 2.5. Preparation of Mycelial Suspension

For mycelial suspension preparation, fungal mycelia were cultured in potato dextrose broth (PDB) at 25 °C with shaking at 150 rpm for 5 days. Then, 7.5 g of mycelia (dry weight) were broken down in the crusher and resuspended in 100 mL of sterile water. Finally, it was diluted to 1 × 10^5^ cfu ml^−1^.

### 2.6. Leaf Colonization Analysis

The strain G3-29 carrying mCherry was shaken in PDB medium for 5 days; hyphae were filtered and prepared into hyphae suspension (1 × 10^5^ cfu mL^−1^) with sterile water. The hyphae suspension was sprayed on healthy kidney bean leaves, and sterile water was used as a control (n = 6). During the 4 days after spraying, the colonization ability of hyphae on kidney bean leaves was observed by a fluorescence microscope.

### 2.7. Pathogenicity Testing

To evaluate the pathogenicity of transformed strains, experiments were conducted using two-week-old kidney bean leaves. We prepared suspensions with a concentration of 1 × 10^5^ cfu mL^−1^ from three transformed strains, pRP27-Eno1-GFP, pRP27-Eno1-ACT, and pRP27-Eno1-SNF. Isolated kidney bean leaves were sprayed, sterile water was used as a negative control, and they were observed for 4 days (n = 6).

### 2.8. RNA Extraction and RT-qPCR

On day 3 of the bioassay, approximately 10 WFTs were randomly sampled. Total RNA was isolated from these insects using the Trizol method and subsequently reverse-transcribed into complementary DNA (cDNA). The expression levels of target genes were then quantified by RT-qPCR, with the elongation factor 1α (*EF1α*) gene serving as an internal reference for normalization.

### 2.9. dsRNA Synthesis

For dsRNA synthesis, gene-specific primers incorporating a T7 promoter sequence were used to amplify template DNAs via PCR. Purified PCR fragments (1 µg each) targeting the *ACT*, *SNF*, and *GFP* genes served as templates for in vitro transcription using the T7 RNAi Transcription Kit (Box 1, Nanjing Vazyme Biotech Co., Ltd., Nanjing, China), following the manufacturer’s instructions. After synthesis, the dsRNA products were treated with a combination of RNase and DNase I at 37 °C for 30 min to remove residual single-stranded RNA and DNA templates. The dsRNA was subsequently purified using magnetic beads to eliminate proteins, salt ions, and unincorporated NTPs (T7 RNAi Transcription Kit Box 2, Nanjing Vazyme Biotech Co., Ltd., Nanjing, China). The dsRNA was applied at a concentration of 500 ng μL^−1^.

### 2.10. WFT Bioassay

Evaluation of the dsRNA delivery system mediated by endogenous fungi in WFTs. First, mycelial suspensions of pRP27-Eno1-GFP, pRP27-Eno1-ACT, and pRP27-Eno1-SNF strains were sprayed onto the surface of two-week-old kidney bean leaves. Four days after spraying, the colonized leaves were transferred to insect observation devices, and larvae or adults were introduced for feeding assays. On the 3rd day after treatment, RT-qPCR was performed, and survival was recorded daily for 4 consecutive days to generate survival curves.

Evaluation of in vitro-synthesized dsRNA against WFTs. Two-week-old kidney bean leaves were selected to make insect observation devices, and larvae or adults were introduced. The concentration of the synthetic dsRNA was adjusted to 500 ng/μL, and it was evenly sprayed onto the body surface of the larvae or adults. On the 3rd day after treatment, RT-qPCR was performed, and survival was recorded daily for 4 consecutive days to generate survival curves.

### 2.11. Statistical Analysis

All data are presented as mean ± standard deviation (SD). Normality and homoscedasticity assumptions were verified, and the data were subjected to an arcsine transformation prior to statistical analysis. Differences between the control and treatment groups were assessed by one-way analysis of variance (ANOVA) followed by Dunnett’s post hoc test for pairwise comparisons. Statistical significance was set at *p* < 0.05 (* *p* < 0.05, ** *p* < 0.01, *** *p* < 0.001, and **** *p* < 0.0001). Sample sizes (n) for each experiment are indicated in the corresponding figure legends. The survival curves were analyzed using the Kaplan–Meier method [29].

## 3. Results

**Figure 1:**
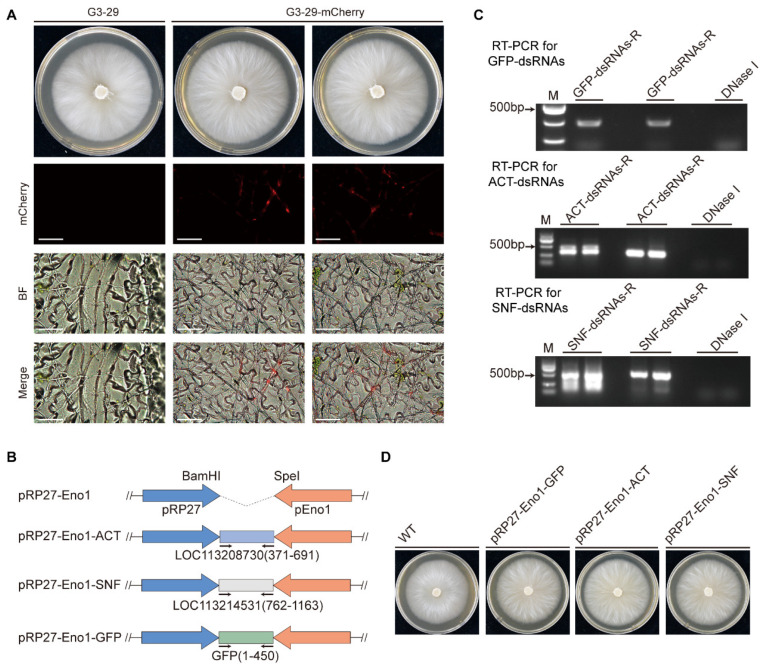
Colonization, vector construction, and characterization of recombinant *F. commune* G3-29 strains. (**A**) Fluorescence microscopy observation of mCherry-labeled G3-29 colonizing kidney bean leaf tissues at 4 days post-inoculation. Scale bar = 100 μm. (**B**) Schematic representation of the dsRNA expression vectors pRP27-ACT-pEno1, pRP27-SNF-pEno1, and pRP27-GFP-pEno1. (**C**) RT-PCR confirmation of dsRNA expression in the transformed G3-29 strains. (**D**) The colony morphology of the three transformed strains, pRP27-ACT-pEno1, pRP27-SNF-pEno1, and pRP27-GFP-pEno1, after 4 days of cultivation on PDA medium (n = 3).

Previous studies have confirmed that the *F. commune* strain G3-29 can successfully colonize the roots, coleoptiles, and leaves of wheat plants [27]. Given that WFTs primarily feed on kidney bean leaves, we investigated whether this strain can also colonize kidney bean leaves. In this study, Two-week-old kidney bean leaves were inoculated with the *F. Commune* strain G3-29 expressing mCherry fluorescent protein. Fluorescence microscopy analysis at 4 days post-inoculation revealed clear red fluorescent signals within the leaf tissue cells, thereby confirming that *F. commune* strain G3-29 could colonize in kidney bean leaves (Figure 1A).

Studies have shown that delivering dsRNA targeting essential genes of insects can achieve effective prevention and control. Therefore, we selected two genes related to growth and development of WFTs, *ACT* and *SNF*, inserted them into the bidirectional promoter vector pRP27-Eno1, and constructed two dsRNA-expressing vectors pRP27-Eno1-ACT and pRP27-Eno1-SNF, with pRP27-Eno1-GFP as a control (Figure 1B). These vectors were transformed into the *F. commune* strain G3-29 via PEG-mediated protoplast transformation. RT-PCR analysis confirmed that all transformed strains successfully expressed the specific dsRNA fragments (Figure 1C). Furthermore, the phenotypes of the two transformed strains showed no significant differences (*p* = 0.6147, *p* = 0.9806) compared to the wild-type and pRP27-Eno1-GFP strains (Figure 1D and Figure S1A). Next, we tested the pathogenicity of three transformed strains (pRP27-Eno1-GFP, pRP27-Eno1-ACT, and pRP27-Eno1-SNF) on kidney bean leaves. After spraying mycelial suspensions of the transformed strains onto detached kidney bean leaves, no significant differences in leaf conditions were observed between the transformed strains and the sterile water control after 4 days (Figure S1B). These results indicate that all three transformed strains are non-pathogenic to bean leaves.

**Figure 2:**
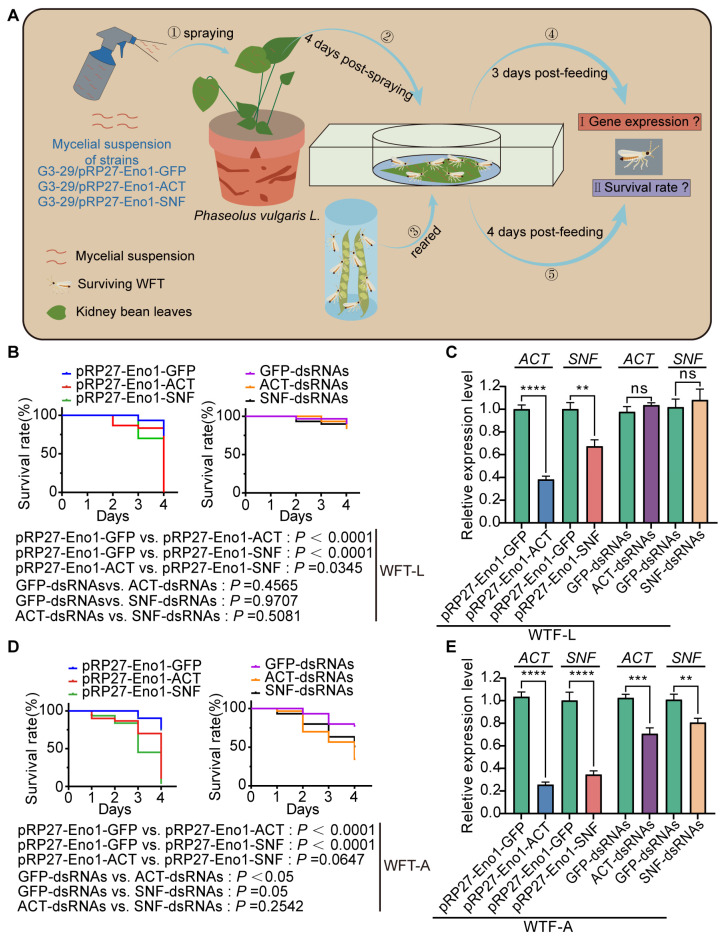
Endophyte-mediated RNAi efficacy against WFTs compared with in vitro-synthesized dsRNA. (**A**) Schematic overview of the bioassay workflow: kidney bean leaves were sprayed with mycelial suspensions of transformed G3-29 strains (expressing ACT-dsRNA, SNF-dsRNA, or GFP-dsRNA). Subsequently, insect feeding tests were conducted on the colonized leaves using first-instar larvae or adults. (**B**) Survival curves of larvae over 4 days after feeding on colonized leaves or on in vitro-synthesized dsRNA. (**C**) The expression levels of the *ACT* and *SNF* genes in larvae 3 days after feeding on colonized leaves or on in vitro-synthesized dsRNA (n = 10 larvae per replicate). (**D**) Survival curves of adult over 4 days after feeding on colonized leaves or on in vitro-synthesized dsRNA. (**E**) The expression levels of the *ACT* and *SNF* genes in adult 3 days after feeding on colonized leaves or on in vitro-synthesized dsRNA (n = 10 adults per replicate). Data represent mean ± SD from three independent experiments. Statistical significance was determined by one-way ANOVA with Dunnett’s post hoc test. The survival curves were analyzed using the Kaplan–Meier method (ns: no significance, ** *p* < 0.01, *** *p* < 0.001, **** *p* < 0.0001).

To assess the efficacy of dsRNA expressed by *F. commune* strain G3-29 against WFTs, insect bioassays were performed on first-instar larvae and adults of WFTs (Figure 2A). The results showed that treatment with the pRP27-Eno1-ACT and pRP27-Eno1-SNF strains significantly reduced (*p* < 0.05) the survival of WFTs compared to the control pRP27-Eno1-GFP strain, with the survival rate dropping below 10% (Figure 2B). RT-qPCR analysis at three days post-treatment revealed that, relative to the pRP27-Eno1-GFP strain control, the expression levels of *ACT* and *SNF* in larvae were significantly reduced (*p* < 0.05) by 63% and 33%, respectively (Figure 2C). In parallel, we evaluated the effects of the same treatment on adults. The results showed that, relative to the pRP27-Eno1-GFP strain control, treatment with pRP27-Eno1-ACT and pRP27-Eno1-SNF strains significantly reduced (*p* < 0.05) the adult survival rate, dropping it to below 10% (Figure 2D). Concurrently, RT-qPCR analysis revealed that the expression levels of *ACT* and *SNF* in adults were significantly reduced (*p* < 0.05) by 74% and 65%, respectively (Figure 2E). For comparison, we synthesized the dsRNAs of the *ACT* and *SNF* genes in vitro (Figure S2). In contrast to the fungus-mediated delivery, in vitro-synthesized dsRNA failed to induce significant mortality (*p* = 0.4565, *p* = 0.9707) or gene silencing (*p* = 0.1088, *p* = 0.4107) in larval stages (Figure 2B,C). Notably, endophytic fungus-mediated dsRNA delivery resulted in superior control of adults compared to the application of in vitro-synthesized dsRNA (Figure 2D,E). Collectively, these bioassay results demonstrate that recombinant *F. commune* G3-29 strains expressing dsRNA targeting the *ACT* and *SNF* genes effectively silence these target genes in both larvae and adults of WFTs.

## 4. Discussion

As a globally significant agricultural pest, WFTs present a concealed feeding habit, a wide range of host species, and an increasingly serious problem of drug resistance, which poses a severe challenge to traditional chemical control strategies [30,31]. In recent years, RNAi technology has emerged as a promising tool for pest management due to its sequence-specific gene silencing mechanism [32]. However, how to achieve efficient and stable delivery of dsRNA under field conditions has always been a core issue. In this study, we constructed a dsRNA expression system based on the endophytic fungus *F. commune* G3-29 and systematically evaluated its efficacy against WFTs. By comparing the effects of endophyte-delivered dsRNA with those of in vitro-synthesized dsRNA, we demonstrated the superior performance of this fungal delivery system, providing new experimental evidence for the PPGS strategy. Our findings establish that the PPGS system centered on *F. commune* G3-29 effectively induces target gene silencing and controls WFTs, offering a novel and sustainable approach for pest management by integrating the colonization capacity of endophytic fungi with the sequence specificity of RNAi.

Regarding target gene selection, our study confirmed that targeting the *ACT* gene and the *SNF* gene resulted in significant lethal effects on WFTs. Actin, as a core component of the cytoskeleton, plays a critical role in cell division, growth, and development. Thus, impairment of its function severely disrupts these essential physiological processes [33,34]. Similarly, the SNF7 protein is a highly conserved core component of the ESCRT-III complex, which is indispensable for endosomal sorting, membrane remodeling, and cell division in eukaryotes [35,36]. Our results are consistent with those reported by previous researchers [15], further supporting the validity and robustness of these target genes for RNAi-based pest control.

In this study, the system of endophytic fungi delivering dsRNA showed significant control efficacy against both adults and larvae, whereas in vitro-synthesized dsRNA had a significant effect only on adults but not on larvae, which is consistent with the report by Han [37]. After WFT fed on the dsRNA expressed by endogenous fungi, the expression levels of the target genes in the larvae were significantly downregulated. However, the in vitro-synthesized dsRNA failed to induce significant mortality or gene silencing in the larvae (Figure 2B,C). Studies have shown that “naked” in vitro-synthesized dsRNA is highly susceptible to degradation by environmental factors and has reduced bioavailability when applied to the leaf surface [38]. Additionally, the mouthpart structure and feeding behavior of thrips larvae may affect their efficiency in taking up dsRNA deposited on the surface [39]. Thrips feed using a “piercing–sucking” method, where their mouthparts first pierce the plant epidermal cells, secrete digestive enzymes, and then ingest the cell contents [39,40,41]. During this process, if the dsRNA is only attached to the leaf surface and does not enter the plant cells, it may be difficult for the larvae to effectively ingest it [42]. In contrast, the endophytic fungal strain G3-29 successfully colonizes the internal tissues of bean leaves (Figure 2A) and continuously produces target dsRNA. Subsequently, these dsRNAs are processed into sRNAs and secreted into the plant, providing a stable and continuous source of sRNAs for feeding WFTs. This approach effectively avoids the degradation issues associated with in vitro application of dsRNA. Subsequently, we can further track the target sRNAs in plants and WFTs using small RNA sequencing (sRNA-seq).

Our approach has advantages over established RNAi strategies. Firstly, unlike HIGS, our system does not rely on plant transgenesis, thereby avoiding the regulatory hurdles and public acceptance concerns associated with genetically modified crops [17]. Secondly, compared with SIGS, it circumvents the high cost of in vitro dsRNA synthesis and the environmental instability of naked dsRNA, which is prone to degradation by ultraviolet radiation, rainfall, and nucleases during spraying [43]. By colonizing plant tissues, endophytic fungi provide sustained in-plant production of dsRNA, ensuring stable protection for the host plants [44]. Although we have not systematically studied the colonization of strain G3-29 in kidney bean plants, our previous research has shown that this strain can successfully colonize the root, stem, and leaf tissues of wheat [28].

However, this study has certain limitations, and further research is needed to evaluate the potential non-target effects, environmental persistence, horizontal transfer risks, and pathogenicity to other plant species associated with introducing a transgenic endophytic fungus into plant tissues under field conditions. Meanwhile, the resistance gene in strain G3-29 will be knocked out in subsequent studies to mitigate the potential risk of horizontal transfer. Additionally, while the strain G3-29 was confirmed to be non-pathogenic to kidney bean leaves in this study, its colonization capacity in a broader range of host plants commonly infested by WFTs, such as pepper, tomato, and ornamental crops, remains to be explored. In the future, our studies should also explore the colonization capacity of *F. commune* G3-29 in a broader range of host plants and extend to other target genes and pest species for broader applications. Overall, PPGS offers a promising tool for the sustainable management of polyphagous insect pests affecting diverse host plants.

## Data Availability

The data that support the findings of this study are available in the Supplementary Material of this article.

## Supplementary Materials

The following supporting information can be downloaded at: https://www.mdpi.com/article/10.3390/jof12040291/s1, Figure S1: Analysis of colony diameters of recombinant *F. commune* G3-29 strains and evaluation of its pathogenicity on kidney bean leaves; Figure S2: The in vitro-synthesized dsRNAs targeting ACT, SNF, and GFP were analyzed by gel electrophoresis to confirm their integrity and size; Table S1: Primer sequences used in this study.
