# Supplementary material for: Endophytic Fusarium commune G3-29-Mediated dsRNA Delivery for Efficient Control of Western Flower Thrips
Source: J Fungi (Basel). 2026 Apr 18;12(4):291. doi: 10.3390/jof12040291 (PMC13117232; doi:10.3390/jof12040291)
Supplement: Supplementary file 1 [file jof-12-00291-s001.zip › jof-4212487-supplementary.pdf]

Supporting Information for

Brief Report

Endophytic *Fusarium commune* G3-29-Mediated dsRNA Delivery for Efficient Control of Western Flower Thrips

Xueyuan Sheng [1,†], Yanfei Wang [2,†], Chang Chen [2], Chao Ma [2], Shuangchao Wang [2], Endong Wang [2], Yan Zhao [1,*] and Lihua Guo [2,*]

[1] Key Laboratory of National Forestry and Grassland Administration on Native Grass Breeding, College of Grassland Science, Inner Mongolia Agricultural University, Hohhot 010018, China

[2] State Key Laboratory for Biology of Plant Diseases and Insect Pests, Institute of Plant Protection, Chinese Academy of Agricultural Sciences, Beijing 100193, China

* Correspondence: zhaoyannmg@imau.edu.cn (Y.Z.); guolihua@caas.cn (L.G.); Tel.: +86-13948416835 (Y.Z.); +86-13681473285 (L.G.); Fax: +86-0471-4301371 (Y.Z.); +86-010-82105928 (L.G.)

† These authors contributed equally to this work.

This PDF file includes:

Figure S1 Analysis of colony diameters of recombinant *F. commune* G3-29 strains and evaluation of its pathogenicity on kidney bean leaves.

Figure S2 The in vitro-synthesized dsRNAs targeting *ACT*, *SNF*, and *GFP* were analyzed by gel electrophoresis to confirm their integrity and size.

Table S1 Primer sequences used in this study.

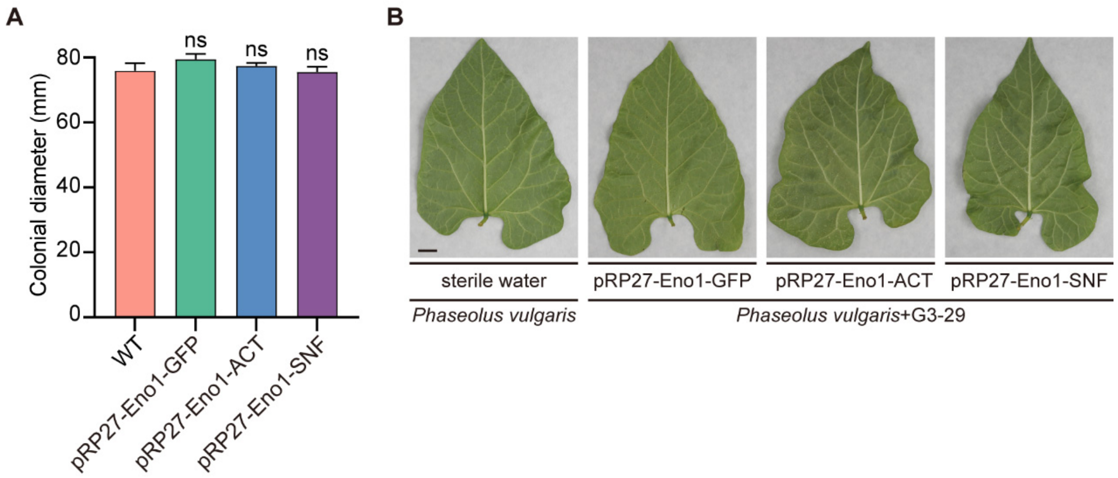

Figure S1. Analysis of colony diameters of recombinant *F. commune* G3-29 strains and evaluation of its pathogenicity on kidney bean leaves.

(**A**) Analysis of colony diameters of the three transformed strains: pRP27-ACT-pEno1, pRP27-SNF-pEno1, and pRP27-GFP-pEno1 (n=3). (**B**) pathogenicity evaluation of the recombinant *F. commune* G3-29 strains on kidney bean leaves.

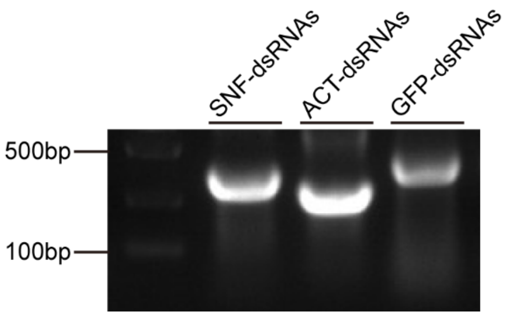

Figure S2. The in vitro-synthesized dsRNAs targeting *ACT*, *SNF*, and *GFP* were analyzed by gel electrophoresis to confirm their integrity and size.

Table S1 Primer sequences used in this study.

Primers	Sequence（5′ to 3′）	Use of PCR products
GFP-dsRNA-F	ATGGTGAGCAAGGGCGAG	To clone the 450 bp *GFP* gene sequence
GFP-dsRNA-R	GTTGTGGCTGTTGTAGTTGTACTC	
ACT-dsRNA-F	CGCGGATCCCCTGAAGTACCCCATTGAAC	To clone the 321bp *ACT* gene sequence
ACT-dsRNA-R	GGACTAGTTGGGGCAGAGCATAACCTTCA	
SNF-dsRNA-F	CGCGGATCCAGCTTGTGGTATGGAAAATGTA	To clone the 402bp *SNF* gene sequence
SNF-dsRNA -R	GGACTAGTCGAATTCTTGCCAATAAAACTT	
qPCR-EF1α-F	AAGGAACTGCGTCGTGGATA	To RT-qPCR analysis of *EF1α* expression in WFT (as reference gene)
qPCR-EF1α-R	AGGGTGGTTCAGGACAATGA	
qPCR-ACT-F	GTCTGGTGCTACCATCGCTT	To RT-qPCR analysis of *ACT* expression in WFT
qPCR-ACT-R	CTTGCACATACCGGACCCAT	
qPCR-SNF-F	GGGTGCTTCGCTTTCAACTC	To RT-qPCR analysis of *SNF* expression in WFT
qPCR-SNF-R	CTCTGTCACGAAAAGGGGCA	
T7-dsGFP-F	TAATACGACTCACTATAGGGATGGTGAGCAAGGGCGAG	To synthesize GFP-dsRNAs
T7-dsGFP-R	TAATACGACTCACTATAGGGGTTGTGGCTGTTGTAGTTGTACTC	
T7-dsACT-F	TAATACGACTCACTATAGGGCCTGAAGTACCCCATTGAACACG	To synthesize ACT-dsRNAs
T7-dsACT-R	TAATACGACTCACTATAGGGTGGGGCAGAGCATAACCTTCA	
T7-dsSNF-F	TAATACGACTCACTATAGGGAGCTTGTGGTATGGAAAATGTACCT	To synthesize SNF-dsRNAs
T7-dsSNF-R	TAATACGACTCACTATAGGGCGAATTCTTGCCAATAAAACTTGAACA	
